# Comparison between Vapocoolant Spray and Eutectic Mixture of Local Anesthetics Cream in Reducing Pain during Spinal Injections

**DOI:** 10.1155/2018/5050273

**Published:** 2018-09-09

**Authors:** Riyadh Firdaus, Besthadi Sukmono, Annemarie Chrysantia Melati, Berial Dewin Marzaini

**Affiliations:** Department of Anesthesiology and Intensive Care, Cipto Mangunkusumo National Central Hospital and Faculty of Medicine, Universitas Indonesia, Jakarta 10430, Indonesia

## Abstract

**Background:**

Eutectic mixture of local anesthetics (EMLA) cream is often used for local anesthesia during spinal injections. However, this agent has delayed onset of action while vapocoolant spray serves more advantages. The vapocoolant spray containing ethyl chloride has fast onset and is safe, low cost, and widely available. This study aimed at comparing the effectiveness of vapocoolant spray and EMLA cream in reducing pain for spinal injections.

**Methods:**

This was an experimental study on 94 subjects with 47 subjects treated with EMLA cream and 47 subjects treated with vapocoolant spray. The effectiveness of anesthesia was assessed by using Numeric Pain Rating Scale (NPRS) and patient movement during the surgery.

**Results:**

This study found that the pain scale was NPRS 0 (0–3) for the EMLA group and NPRS 0 (0–4) for the vapocoolant spray group. There was no significant difference between two groups for pain scale according to the Mann–Whitney *U* test. For patient movement, the movement was reported only in one (2.1%) patient in the EMLA group and one (2.1%) patient in the vapocoolant spray group. Based on Fisher's test, there was no significant difference between the two groups for patient movement.

**Conclusions:**

Both EMLA cream and vapocoolant spray were equally effective in reducing pain during spinal injection. There was no difference in degree of pain reduction and patient movement between the EMLA cream group and the vapocoolant spray group during spinal injection.

## 1. Background

Pain during any medical procedures, including spinal anesthesia, should be managed carefully as this would affect the perception and comfort of the patients [1, 2]. Kim et al. mentioned that pain during spinal injections had mean pain scale of 3.9 based on Numeric Pain Rating Scale (NPRS). Additionally, the diameter of the needle was correlated with the pain during injections: 32 G needle generated pain in 31% of patients, 27 G needle generated pain in 53% of patients, and 23 G needle generated pain in 63% of patients [3]. Reduced pain during spinal injection would increase the quality of anesthesia. Additionally, the patient would have positive experience during the anesthesia procedure.

Local anesthetic agents may be administered to relieve the pain during any injections. Anesthetic agents often used are eutectic mixture of local anesthetics (EMLA) cream, ethyl chloride, nonsteroidal anti-inflammatory drugs (NSAIDs), lidocaine, and opioid [4–6]. EMLA cream is often used to decrease the pain during spinal injections. However, this agent has longer onset of action, up to 30 minutes, before the peak of action [7].

The vapocoolant spray contains ethyl chloride which serves as topical anesthetic agents. The vapocoolant spray generates cooling effect on the skin surface. There are many advantages of this agent, including fast onset of action, its safety profile, and low cost, and it is widely available [8]. The mechanism of action is that the vapors create sudden decrease of the skin temperature and disrupt the ion channel activation. Therefore, this would impair the pain reception [9]. This agent is often used in minor medical procedures, including arterial/venous cannulation [10].

Past studies have mentioned the wide indications of vapocoolant spray for many medical procedures. Celik et al. have compared the administration of vapocoolant spray, EMLA cream, and placebo cream during venous cannulation among patients undergoing hemodialysis. The result showed that both vapocoolant spray and EMLA cream significantly decreased the pain intensity during injections [11].

Based on the preliminary study conducted during February 2016 in Cipto Mangunkusumo Hospital, there were 94.3% of respondents who had movements during spinal injections by using 27 G needle. Additionally, there were 5.7% respondents with pain score of 5, 25.7% respondents with pain scale of 3, 51.4% respondents with pain scale of 2, and 17.1% respondents with pain score of 1. The mean pain score for all respondents undergoing spinal injections was 2.3.

In Indonesia, currently there is no study regarding the use of vapocoolant spray as local anesthetic agent during spinal injections. However, this agent is cheaper and widely available in the daily practice in comparison with EMLA cream. This study aimed to compare the effectiveness between vapocoolant spray and EMLA cream as the local anesthetic agent during spinal injections.

## 2. Methods

### 2.1. Research Design

This was an experimental study conducted in a stereotactic operating room of Cipto Mangunkusumo Hospital from October 2016 to January 2017. Research subjects were divided into two groups with administration of vapocoolant spray containing ethyl chloride for the treatment group and EMLA cream for the control group.

### 2.2. Inclusion and Exclusion Criteria

Inclusion criteria were adult subjects over the age of 18 undergoing first brachytherapy procedure and planned for spinal anesthesia and having physical status of the American Society of Anesthesiologist (ASA) I–III. Exclusion criteria were subjects with allergy to anesthetic drugs, hemodynamically unstable, contraindications to spinal injection, unwilling to sign informed consent, and current consumption of psychiatric drug. Drop-out criteria were subjects with allergic reaction during injection, refractory hypotension (decreased blood pressure >20%) despite of administration of ephedrine, and refractory desaturation (peripheral oxygen saturation <92%) despite of positive ventilation pressure.

### 2.3. Research Protocol

There were 94 subjects with 47 subjects for each group consecutively. Following the ethical clearance, and registered in clinicaltrials.gov (NCT 03134391), research subjects were randomized by using block randomization into control and experimental group. All subjects had 22 G intravenous cannula with ringer acetate fluid administration. Mean arterial pressure (MAP), pulse rate, respiratory rate, and peripheral capillary oxygen saturation (SpO_2_) were recorded. Patients were positioned in sitting position with the head flexed and back arched posteriorly. Spinal injection site was identified in the L4-5 intervertebral space.

In the EMLA group, research subjects received 2 ml of EMLA cream containing a mixture of 2.5% lidocaine and 2.5% prilocaine in oil/water emulsion and further covered by clear dressing for 45–60 minutes. In the vapocoolant spray group, the patient received a spray from a vapocoolant pressure pack with a distance of about 10 cm from the surface for two seconds and at least 10 seconds for the vapocoolant spray to vaporize.

Spinal injection was conducted by using 27 G spinal needle with pointed tip with the bevel parallel to the durafiber. Spinal injection was conducted by a final year anesthesiologist resident. Pain intensity was measured by using NPRS, and patient movement was recorded to measure the success of anesthesia. Vital signs were observed every three minutes for the first 15 minutes and every five minutes until completion of the procedure.

### 2.4. Statistical Analysis

Data analysis was performed by using the Statistical Package for the Social Sciences (SPSS) for Windows version 20.0. NPRS score and patient movement was analyzed by using the independent *T*-test and chi-squared test if data had normal distribution and Mann–Whitney *U* test and Fisher's exact test if data had nonnormal distribution.

## 3. Results

**Figure 1:**
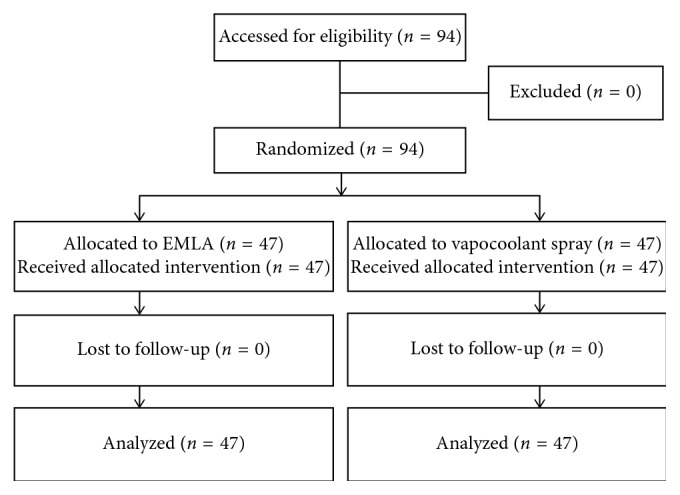
Study flow according to the Consolidated Standards of Reporting Trials (CONSORT) diagram.

There were no subjects that dropped out during the study (Figure 1). Baseline characteristics of study subjects are shown in Table 1. Demographic data were comparable between the EMLA cream group and the vapocoolant spray group. There was no significant difference in anthropometrical status such as weight, height, and body mass index between the two groups.

Data on pain intensity using NPRS and patient movement in both groups are shown in Tables 2 and 3. There was no significant difference in both groups for patient intensity and also movement.

**Figure 2:**
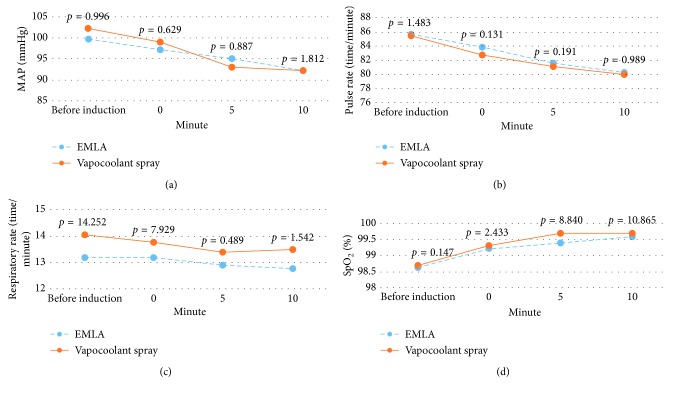
Analysis of hemodynamic profile. (a) Mean arterial pressure analysis by time. (b) Pulse rate analysis by time. (c) Respiratory rate analysis by time. (d) Peripheral capillary oxygen saturation analysis by time.

There were no allergic reactions occurred during the study. There was no occurrence of desaturation/apnea events, bradycardia, nausea, vomiting, respiratory depression, hypotension, and agitation in both groups. Analysis of hemodynamic profile (MAP, pulse rate, respiratory rate, and SpO_2_) according to time is shown in Figure 2. Based on unpaired *T*-test results, there were no significant differences for hemodynamic profile in the form of MAP, pulse rate, respiratory rate, and SpO_2_.

## 4. Discussion

This study aimed to compare the effectiveness of vapocoolant spray and EMLA cream in reducing pain for spinal injections. Based on pain assessment in this study generated, there was no statistically significant pain scale difference between these two. This was similar with previous study by Cohen et al., in which found there were no significant differences in the duration of crying, pain-related behavior, and pain based on child-scored faces scale and parent-scored faces scale [12]. Conversely, Celik et al. found that the pain intensity based on NPRS with EMLA cream is significantly lower than that with vapocoolant spray. This contradictory result might be due to differences in study population. In a study by Çelik et al., as many as 24.4% of subjects had diabetes mellitus as the etiology of renal failure; thus, it may also be accompanied by peripheral neuropathy [11]. Moon et al. also showed different results, in which the vapocoolant spray was more effective in reducing pain compared with topical anesthetic cream for needle electromyography in the extremities region [13].

The ability to reduce pain following spinal injections between EMLA cream and vapocoolant spray was comparable. Both anesthetic agents might be utilized to reduce pain during regional anesthesia, including spinal injections. However, this study could not generate the similar finding for other medical procedures that required larger diameter of cannula. Further study should be conducted with different medical procedures.

Allergic reaction and other adverse event related with the procedure, such as desaturation/apnea events, bradycardia, nausea, vomiting, respiratory depression, hypotension, and agitation, were recorded in this study. However, there was no such finding in this study. The literature showed that the side effects associated with vapocoolant spray and EMLA cream anesthetic were quite rare. Çelik et al. mentioned only one subject suffered from allergic reaction following the administration of EMLA cream [11]. Any topical anesthetic agents should be absorbed locally. Therefore, it has minimal to no systemic effects. This study generated no significant hemodynamic profile differences between two groups.

This study found that in both groups, majority of patients moved during spinal injection similar to the preliminary study in which more than 90% of patients moved during the spinal injection. Therefore, the presence of local anesthetic during spinal injection did not affect any movement during spinal injection [14]. Movement during spinal injection might be contributed by many factors, such as nervousness, jitteriness, or fear. This movement did not necessarily mean that the patient felt any pain during injection.

Both anesthetic agents were widely available in the daily practice with excellent safety profile. EMLA cream is commonly used for local anesthesia in spinal injection. This study has shown that with regard to the ability to reduce pain during spinal injection, there was no significant difference between these two agents. However, one of the disadvantages of EMLA cream is its delayed onset of action; the patients should wait up to 60 minutes before injection after the administration of the EMLA cream. On the contrary, the vapocoolant spray showed that it has a rapid onset of action in which the patient only needed to wait for ten seconds before injection. This difference was clinically significant so that the vapocoolant spray was more suitable for any procedure that required shorter preparation time, such as emergency procedure. Additionally, both surgeons and patients did not have to wait an hour before starting the anesthesia procedure. Therefore, this was the main advantage of the vapocoolant spray.

There was some limitation in this study. Due to the preparation of both agents, it was difficult to do blinding for both researcher and subjects. Additionally, pain scale by using NPRS was a subjective assessment even though pain perception is subjective for each patient. However, this could create bias in this study. This study did not measure the difference in duration of the spinal anesthesia procedure. Reduced pain might affect the duration as well as the success rate of the spinal injection.

## 5. Conclusion

Administration of EMLA cream and vapocoolant spray was equally effective in reducing pain during spinal injection. There was no difference in terms of pain reduction between the EMLA cream group and the vapocoolant spray group during spinal injection.

## Figures and Tables

**Table 1:** Baseline characteristic of both groups.

Characteristics	EMLA cream *N*=47	Vapocoolant spray *N*=47	*p* value
Age (years)	52.43 ± 10.37	53.81 ± 8.78	0.488^a^

Occupation			0.144^b^
Housewife	33 (70.2%)	39 (83.0%)	—
Non-housewife	14 (29.8%)	8 (17.0%)	—

Education			0.539^b^
None	3 (6.4%)	3 (6.4%)	—
Elementary school	18 (38.3%)	16 (34.0%)	—
Junior high school	8 (17.0%)	7 (14.9%)	—
Senior high school	14 (29.8%)	11 (23.4%)	—
University	4 (8.5%)	10 (21.3%)	—

Weight (kilogram)	55.70 ± 14.23	54.52 ± 10.11	0.645^a^

Height (centimeter)	150.34 ± 6.01	151.0 ± 682	0.619^a^

Body mass index	23.56 (14.79–52.07)	23.68 (17.10–36.89)	0.922^c^

ASA physical status			1.000^d^
I	0 (0%)	2 (4.3%)	—
II	45 (95.7%)	43 (91.5%)	—
III	2 (4.3%)	2 (4.3%)	—

Diagnosis			1.000^d^
Cervical cancer	45 (95.7%)	46 (97.9%)	—
Vagina cancer	1 (2.1%)	1 (2.1%)	—
Endometrium cancer	1 (2.1%)	0 (0%)	—

Numerical data were presented as mean ± standard deviation if distribution was normal and median (minimum-maximum) if distribution was not normal; categorical data were presented as *N* (percentage). ^a^Independent *T*-test; ^b^chi-squared test; ^c^Mann–Whitney *U* test; ^d^Kolmogorov–Smirnov test.

**Table 2:** Comparison of pain intensity during spinal injection between the EMLA cream group and the vapocoolant spray group.

NPRS^a^	EMLA cream (*N*=47)	Vapocoolant spray (*N*=47)	*p* value
NPRS 0	30	25	1.000^b^
NPRS 1	13	17	
NPRS 2	3	4	
NPRS 3	1	0	
NPRS 4	0	1	

^a^NPRS, numeric pain rating scale; ^b^Mann–Whitney *U* test, significant if *p* value < 0.05.

**Table 3:** Comparison of patient movement during spinal injection between the EMLA cream group and the vapocoolant spray group.

Group	*N*	Patient movement		*p* value
		No movement	Movement	
EMLA cream	47	46 (97.9%)	1 (2.1%)	1.000^a^
Vapocoolant spray	47	46 (97.9%)	1 (2.1%)	

^a^Fisher's exact test, significant if *p* value < 0.05.

## Data Availability

The data used to support the findings of this study are available from the corresponding author upon request.
